# Longitudinal effects of emotion awareness and regulation on mental health symptoms in adolescents with and without hearing loss

**DOI:** 10.1007/s00787-021-01900-9

**Published:** 2022-02-22

**Authors:** Adva Eichengreen, Evelien Broekhof, Yung-Ting Tsou, Carolien Rieffe

**Affiliations:** 1 Department of Developmental and Educational Psychology, Institute of Psychology, Leiden University, Leiden, The Netherlands; 2 Center for Disability Studies, The Paul Baerwald School of Social Work and Social Welfare, The Hebrew University of Jerusalem, Jerusalem, Israel; 3 The E. Richard Feinberg Department of Child and Adolescent Psychiatry, Schneider Children’s Medical Center, Petah Tikva, Israel; 4 Department of Human Media Interaction, Faculty of Electrical Engineering, Mathematics and Computer Science, University of Twente, Enschede, The Netherlands; 5 Department of Psychology and Human Development, Institute of Education, University College London, London, UK

**Keywords:** Longitudinal study, Emotion awareness, Emotion regulation, Internalizing and externalizing symptoms, Deaf and hard of hearing

## Abstract

Emotion awareness (EA) and regulation (ER) are each known to associate with mental health symptoms, yet there is a paucity of longitudinal studies examining them jointly during adolescence. Furthermore, little is known about these skills and their relations in deaf and hard-of-hearing (DHH) adolescents, who are at risk for reduced emotion socialization and for more mental health symptoms. This longitudinal study examined the development and unique contributions of EA (emotion differentiation, emotion communication and bodily unawareness) and ER (approach, avoidance and worry/rumination) to internalizing and externalizing symptoms in adolescents with and without hearing loss. Using self- and parent's reports, we assessed 307 adolescents (age 9–15) three times over 18-month period. We found stability over time in development of EA and avoidance ER, increase in approach ER and decrease in worry/rumination. High levels and increases over time in two aspects of EA, emotion differentiation and communication, and in approach and avoidance ER were related to decreases in depressive symptoms. An increase in approach ER was also related to a decrease in anxiety symptoms. Yet, low levels or decreases in worry/rumination were related to decreased levels of depressive, anxiety and externalizing symptoms. Hearing loss did not moderate any of the variables or relations tested. Preliminary tests suggested heterogeneity within the DHH group according to educational placement, language abilities and parental education level. Overall, findings pointed at unique contributions of EA and ER to mental health development, suggesting that DHH adolescents, especially in mainstream schools, do not differ from their hearing peers in their emotion awareness and regulation.

## Introduction

The contribution of emotion awareness (EA) and emotion regulation (ER) to the prevention or generation of psychopathology has long been studied, both in research and in clinical practice [1]. Identifying one's own emotions, relating them to the triggering situation, and selecting and implementing adaptive regulatory strategies, enable individuals to resolve emotion-evoking situations or to cope with negative emotions in a way that prevents the generation of psychopathological symptoms [1, 2]. While most research has been cross-sectional and studied EA and ER separately from each other, this study applied a longitudinal design to examine the unique contributions of EA and ER to internalizing and externalizing symptoms together. Further, while EA and ER skills are known to be acquired through social learning [3], very little is known about these processes in children and adolescents who have less access to their social environment. Such are deaf and hard-of-hearing (DHH), who are at risk for reduced emotion socialization [e.g., 4] and mental health development [e.g., reviews at 5, 6]. This study is the first to examine the longitudinal effects of EA and ER on mental health development in adolescents with hearing loss, compared to hearing peers.

Awareness of one's own emotions is considered a prerequisite for adaptive regulation [7]. This core feature in our emotional development is defined as an *attentional* process that enables individuals to identify emotion experiences, differentiate between emotions and locate their antecedents [2, 7, 8]. The ability to identify the cause of the emotions requires directing one's focus from internal bodily arousal to the external environment. It has been shown that a greater ability to identify and differentiate between emotions is related to relative unawareness of bodily sensations during the emotion experience [9]. Besides attentional aspects, EA includes *attitudinal* aspects, such as the extent to which one tends to, or thinks that one should, communicate emotions to other people [9, 10].

EA skills are particularly important during the transition to adolescence, a period characterized by new stressors and a greater vulnerability to mental health symptoms. During this period intense physiological, cognitive, and emotional changes emerge, all of which may lead to increased social and emotional difficulties [11, 12]. At the same time, development in meta-cognitive skills allows adolescents to develop more complex and self-reliant emotion skills [13, 14]. Cross-sectional studies in adolescents have clearly indicated that EA skills, including emotion differentiation, emotion communication, and bodily unawareness, are negatively associated with internalizing symptoms such as depression and anxiety [8–10, 15–19]. Longitudinal studies have shown that emotion differentiation predicted less depressive symptoms over time [2, 8, 16, 20]. The link between EA and externalizing problems has received much less attention, yet there is cross-sectional evidence of a negative association between emotion differentiation and externalizing symptoms such as conduct problems in adolescents [19, 21].

Besides awareness to one's emotions, being able to regulate them is crucial for mental health and includes inducing a change in the generation of one's emotion, the experience of that emotion, or the way the person reacts to and acts on the emotion experience [1]. A variety of regulation strategies have been studied in relation to mental health symptoms in children and adolescents [e.g., review at 13]. One central adaptive category of strategies is approaching the stressor with the aim of modifying it, for instance by actively thinking about how to solve it. Approach strategies are negatively associated with internalizing symptoms such as depression and anxiety [13, 22, 23], and with externalizing symptoms such as aggression and conduct disorder [13]. In addition, cognitive avoidance from the stressor can also be used to regulate internal stress, such as minimizing the importance of the stressor or distracting oneself to a positive cognition/activity. Avoidance strategies were found to negatively relate to depression in children and adolescents [24] and are effective especially when the stressor is conceived as uncontrollable [25], such as in the experiences of ethnic minority adolescents [25], or adolescents with autism [26]. However, longitudinal evidence of the effectiveness of these adaptive regulation strategies is lacking, with only few studies showing no effect of approach strategies on mental health over time [13, 23].

In contrast, rumination has been clearly identified as a maladaptive internalizing regulation strategy, and was conceptualized as a transdiagnostic factor explaining multiple forms of psychopathology [review at 27]. Concurrent and prospective studies in adolescents have shown that the tendency to dwell on the problem by repetitively worrying about it or thinking about its negative meanings is associated with depression and anxiety and predicts them over time [8, 27]. Recently, studies in early adolescents have shown that worry/rumination also underlies externalizing symptoms in boys, such as aggressive or disruptive behavior and their comorbidity with internalizing symptoms [28, 29]. Rumination over a provocative trigger may underlie a transition between a depressed or anxious mental state to an aggressive behavior. In turn, provocative or aggressive behaviors can lead to negative social or academic consequences, which by themselves can increase engagement in rumination [28].

Alongside the significant body of research on EA and ER skills in adolescents, only few studies have examined them in tandem, despite the strong association between them [30]. Among the studies which tested the contributions of both emotion awareness and regulation to mental health development composites scores were used, which either unified EA with ER [31, 32], or measured overall levels of EA and ER with no distinction between specific EA/ER skills [20, 33]. Therefore, more research is needed on the contribution of EA and ER to mental health while controlling for the effect of each other, and while disentangling to specific awareness and regulation strategies.

Moreover, there is lack of studies examining the development of EA and ER and their contributions to mental health in adolescents who are at risk for less exposure to interactions within their family and social environments. Identifying and labeling one's emotion experiences and developing strategies to regulate them depend to a large extent on linguistic, social, and cultural learning [3, 7]. Both parents and peers play a crucial role in these processes throughout childhood and adolescence. Emotion socialization by parents, peers and other social agents occurs directly, by responding to or guiding the child's emotion expressions, or indirectly by modeling attitudes and skills, which the child observes or overhears [3, 12]. Very little is known about the development of EA and ER in children and adolescents with compromised access to surrounding social interactions. Such are DHH children, who are often born to hearing parents and are raised in auditory-verbal environments in which their access to communication is limited [4]. Difficulties to follow social interactions may make it harder for DHH children and adolescents to infer about the socio-emotional meanings of interactions and learn from them. Overprotectiveness of parents and educators and a reduced discourse on mental states may also result in insufficient socio-emotional coaching of DHH children and adolescents [34, 35].

Importantly, DHH children and adolescents display higher rates of mental health problems compared to hearing peers, in respect to both internalizing and externalizing symptoms [35–38 and reviews at 5, 39]. A recent study in preschoolers showed that even though DHH and hearing groups did not differ on average scores, the DHH group presented a greater variance in rates of psychosocial symptoms [40]. These findings call for further understanding of underlying factors explaining variance in mental health development in DHH children and adolescents. So far, studies have focused on factors such as language, speech, and communication skills [5, 6, 38–41] or the presence of additional disabilities such as intellectual disabilities [5, 6, 39, 40]. Much less is known about the role of EA and ER in the development of mental health in DHH children and adolescents, although these skills are affected by social learning and language acquisition [5].

To date, limited studies have examined EA and ER in DHH children and adolescents, and all these studies were cross-sectional. Studies on EA [42, 43] indicated that DHH adolescents showed similar abilities as hearing peers for emotion differentiation in simple contexts, but a lower ability to differentiate emotions when situations involved multiple emotions. As to emotion regulation, preschoolers with cochlear implants (CI) [44, 45], and deaf adolescents [42, 46, 47] were found to express negative emotions more intensively and bluntly compared to hearing peers. They also used problem-focus approach strategies less often [46]. Alternatively, compared to hearing peers, use of approach strategies was experienced by DHH adolescents as less effective in decreasing negative emotion arousal in themselves and in their partners [42]; presumably due to blunt emotion expression and less attention to the partners' perspectives [47]. In addition, compared to hearing peers, DHH children and adolescents made less use of avoidant self-distraction strategies to calm down and were less successful in thinking about ways to recover and be happy again in hypothetical scenarios [42, 44]. Wiefferink and colleagues [44] also suggested that, compared to hearing peers, young DHH kids may benefit more from self-distraction regulation strategies, as the negative relation between self-distraction and externalizing behavior problems was stronger for this group.

## The present study

The goal of this study was twofold. First, we explored the unique longitudinal contributions of EA and ER to internalizing and externalizing symptoms when examined simultaneously. Second, we examined whether there were differences between adolescents with and without hearing loss in baseline levels and in developmental trajectories of EA and ER and in the longitudinal contributions of EA and ER to mental health symptoms. Questionnaires were administered to 9–15-year-olds with and without hearing loss and their parents on three occasions, with a 9-month interval in between. Emotion awareness was investigated through differentiation of one's own emotions, bodily unawareness, and emotion communication. Emotion regulation was investigated through adaptive regulation strategies (approach, avoidance) and maladaptive worry/rumination. Internalizing problems were examined through depressive and anxiety symptoms, and externalizing problems were examined through symptoms of attention deficit hyperactivity, oppositional defiant, and conduct disorders.

Based on cross-sectional studies [44, 46], we expected the DHH group to present lower baseline levels of EA and ER, compared to the hearing group. Next, we examined in both groups the developmental trajectories of EA and ER. Previous cross-sectional research, which compared between different age groups, has shown mixed findings regarding age-related development of EA [8, 12, 19] and ER [14, 48] skills. Due to the limited longitudinal research in this field [13], this part of the investigation was exploratory. At the third step, we tested the contribution of EA and ER to the development of internalizing and externalizing symptoms. Based on longitudinal data from hearing adolescents [e.g., 2, 27], we expected in both groups to find negative contributions of baseline and change levels of EA and adaptive ER, and a positive contribution of baseline and change levels of worrying/rumination, to the prediction of internalizing and externalizing symptoms. We also expected the negative relation between approach strategies and mental health symptoms to be weaker in the DHH group, based on a previous cross-sectional research which showed that approach strategies and negative emotion arousal were less strongly related in DHH adolescents compared to hearing peers [42]. As for other associations between ER strategies and mental health, no specific hypotheses were made on differences in association magnitude due to lack of empirical studies including DHH adolescents, although a stronger negative relation between avoidant ER and externalizing symptoms was found for a sample with younger DHH children [44]. Finally, as it has been suggested that DHH adolescents in special education present more mental health and psychological difficulties compared to DHH peers in mainstream education [49], we conducted preliminary anlayses to compare between these subgroups in levels of EA, ER and mental health symptoms.

## Method

### Participants

This study was part of a larger project on socio-emotional functioning in children with and without communication difficulties [e.g., 29, 50]. The DHH and hearing samples in this study are the same as in a previous study on bullying and aggression [51]. Part of the cross-sectional data assessed at Time 1 has been previously published [52–54]. A total of 307 adolescents between 9 and 15 years (M = 11.71, SD = 1.45 at Time 1) participated in this study, out of which 80 were DHH and 227 hearing. DHH participants were recruited through hospitals' Otorhinolaryngology departments, speech and hearing centers, special schools for the deaf, and publications at magazines and websites of organizations giving services to DHH youth and their caregivers. Inclusion criteria for the DHH participants was having a prelingual hearing loss of at least 40 dB in the better ear. Hearing participants were recruited nationwide through mainstream schools. The groups did not differ in age (Time 1: *t*(305) = -1.47, *p* = 0.142); gender distribution (*χ*^*2*^(307) = − 0.37, *p* = 0.539), IQ score (*t*(274) = 1.13, *p* = 0.259), language ability (*t*(252) = 0.09, *p* = 0.932), and parental education level (*t*(231) = -0.45, *p* = 0.656). Participants' characteristics are presented in Table 1.

**Table 1:** Demographic characteristics of participants

	DHH	Hearing
No. of participants	80	227
Age in years at T1		
Mean (*SD*)	11.91 (1.62)	11.63 (1.38)
Range	9.17–15.75	9.08–14.75
Gender—*n* (%)		
Male	37 (46.3)	96 (42.3)
Female	43 (53.8)	131 (57.7)
IQ score (*SD*)	10.19 (2.67)	10.61 (2.48)
Language score (*SD*)	10.29 (3.30)	10.32 (2.30)
Parental education level† (*SD*)	3.21 (.72)	3.17 (.66)
Type of education—*n* (%)		
Regular education	48 (60.0)	227 (100.0)
Special education	32 (40.0)	0
Communication mode—*n* (%)		
Dutch Sign Language /Sign Supported Dutch	28 (35.0)	
Spoken Language only	52 (65.0)	
Type of amplification—*n* (%)		
Hearing aid	53 (66.3)	
Cochlear implant (CI)	27 (33.3)	
Hearing loss in best ear—*n* (%)		
40–60 dB	20 (25.0)	
61–90 dB	18 (22.5)	
> 90 dB	36 (45.0)	
Unknown	6 (7.5)	

*DHH* Deaf or heard of hearing, *SD* Standard Deviation, *T* Time

^†^The highest level of education of each parent was categorized on a scale ranging from one to four. Parental education level was calculated by averaging these two scores

**Table 2:** Psychometric properties and mean scores (standard deviations) of study variables at each time point

Parameters	*N* items	Scale	Cronbach’s α	Mean scores (SD)		*t*-value^a^
				DHH	Hearing	
Time 1						
Differentiating emotions	7	1–3	0.78	2.30 (0.45)	2.39 (0.43)	1.47
Bodily unawareness	5	1–3	0.65	1.96 (0.44)	1.84 (0.47)	− 2.01*
Emotion communication	8	1–3	0.76	2.11 (0.37)	2.05 (0.45)	− 1.06
Approach strategies	12	1–3	0.81	2.10 (0.38)	2.13 (0.41)	0.67
Avoidant strategies	12	1–3	0.82	1.86 (0.40)	1.89 (0.39)	0.46
Worry/rumination	10	1–3	0.86	1.92 (0.48)	1.90 (0.46)	− 0.40
Depressive symptoms	26	1–3	0.75	1.38 (0.21)	1.32 (0.19)	− 2.42*
Anxiety symptoms	7	1–4	0.80	1.51 (0.46)	1.41 (0.36)	− 1.47
Externalizing symptoms	40	1–4	0.90	1.40 (0.20)	1.33 (0.23)	− 2.13*
Time 2						
Differentiating emotions	7	1–3	0.74	2.38 (0.42)	2.39 (0.39)	0.07
Bodily unawareness	5	1–3	0.70	2.04 (0.44)	1.86 (0.51)	− 2.74**
Emotion communication	8	1–3	0.74	2.09 (0.39)	2.04 (0.40)	− 1.08
Approach strategies	12	1–3	0.87	2.16 (0.45)	2.24 (0.43)	1.40
Avoidant strategies	12	1–3	0.86	1.95 (0.44)	1.94 (0.41)	− 0.13
Worry/rumination	10	1–3	0.85	1.77 (0.45)	1.86 (0.47)	1.58
Depressive symptoms	26	1–3	0.73	1.36 (0.19)	1.32 (0.18)	− 1.28
Anxiety symptoms	7	1–4	0.77	1.44 (0.38)	1.43 (0.37)	− 0.14
Externalizing symptoms	40	1–4	0.90	1.33 (0.21)	1.33 (0.22)	− 0.02
Time 3						
Differentiating emotions	7	1–3	0.78	2.46 (0.46)	2.38 (0.39)	− 1.37
Bodily unawareness	5	1–3	0.76	1.96 (0.51)	1.83 (0.51)	− 1.75
Emotion communication	8	1–3	0.81	2.20 (0.40)	2.06 (0.44)	− 2.16*
Approach strategies	12	1–3	0.86	2.24 (0.41)	2.27 (0.42)	0.58
Avoidant strategies	12	1–3	0.83	2.01 (0.35)	1.96 (0.39)	− 0.93
Worry/rumination	10	1–3	0.84	1.72 (0.43)	1.85 (0.44)	1.97*
Depressive symptoms	26	1–3	0.74	1.32 (0.19)	1.28 (0.17)	− 1.68
Anxiety symptoms	7	1–4	0.80	1.48 (0.39)	1.41 (0.40)	− 1.06
Externalizing symptoms	40	1–4	0.91	1.34 (0.20)	1.31 (0.23)	− 1.03

*DHH* Deaf or hard of hearing, *SD* Standard Deviation

^a^Pooled results after multiple imputations

^*^p < 0.05; ** *p* < 0.01

### Materials

#### Personal characteristics

*IQ* was assessed using the two nonverbal subtests of the WISC-III (WISC-III^NL^), Block Design and Picture Arrangement [55, 56]. For the Block Design subtest children had to copy geometric designs with plastic cubes. For the Picture Arrangement subtest, children had to arrange cartoons in a specific sequence in order to make logical stories. The obtained scores were converted into age-corrected norm scores, and a mean IQ score was calculated based on the two norm scores (*M* = 10).

*Language ability* was assessed using two subtests of the Clinical Evaluation of Language Fundamentals—Fourth edition [CELF; 57] In the first subtest on understanding spoken paragraphs, participants were presented with spoken information and asked to answer questions about the content. The second subtest, semantic relations, measured the ability to understand sentences involving comparisons, location, serial order, and time relations. Participants listened to a sentence and selected two correct answers from four presented alternatives. The scores were converted into age-corrected norm scores (*M* = 10), and a mean language score was calculated based on the two norm scores (Table 1).

### Predictors

#### Emotion awareness

To measure EA we used the Dutch version of the Emotion Awareness Questionnaire Revised [EAQ-R; 9]. The EAQ is a self-report questionnaire whose original form consists of 30 items representing six subscales that measure EA. For the purpose of this study, we used three scales, consisted of twenty-five items in total: The first scale, *Differentiating Emotions*, referring to the ability to differentiate between emotions and locate their antecedents, consists of seven items (e.g., “*When I am upset, I don’t know if I feel angry or sad*”, reverse scored). The second scale, *Bodily Unawareness*, referring to lack of attention to physiological aspects of the emotion experience, consists of five items (e.g., *"When I am sad, my body feels weak*", reversed scored). The third scale, *Communicating Emotions* (previously the scales Verbal Sharing and Not Hiding Emotions), assessing the tendency to share and explain one's emotions to other people in a verbal way, consists of eight items (e.g., *"I find it difficult to tell others how I feel",* reversed scored). Following the original scale's format participants were asked to rate their responses to EAQ-R items on a 3-point scale (1 = *not true,* 2 = *sometimes true,* 3 = *often true).* Cronbach alpha reliability of the EAQ-R scales has been reported to range between acceptable to good [19]. In this study internal consistencies of the three scales were adequate across the time points (0.65 ≤ α ≤ 0.81).

#### Emotion regulation

To measure adaptive ER, the Dutch version of the Coping Scale [58] was administered to indicate which ER strategies would be used in hypothetical problematic peer scenarios. The Coping Scale [23] consists of six ER strategies, which fall into three subscales. We used the Approach Scale (Problem Solving and Seeking Social Support subscales) and Avoidant Scale (Distraction and Trivialising subscales), each consisted of 12 items. Approach strategies involved approaching the stressor to calm down (e.g., “*I try to think of different ways to solve the problem"* and “*I ask someone in my family for advice*”), whereas avoidant strategies involved creating distance from the emotion-evoking situation to calm down (e.g., *“I do something else to help me forget about it”* and “*I tell myself it doesn't matter*”). Participants were first instructed to imagine a problematic scenario with a peer. Then they answered how often they would use a certain strategy by rating each statement on a 3-point scale (1 = *almost never,* 2 = *sometimes,* 3 = *often*). The scales were reported to have good reliability [58]. The internal consistencies of approach and avoidant scales in this study were good across the three measurements (0.81 ≤ α ≤ 0.87).

To measure maladaptive ER, we used the Worry/rumination questionnaire for children [29, 59]. This 10-item self- report assesses the tendency to dwell on the problem instead of solving it or coping adaptively with its emotional impact. Example items are *"When I have a problem, I can't stop thinking about it"* and *"When I make a mistake, I am worried about what might happen"*. Items were rated on a 3-point scale ranging from 1 = not true to 3 = often true. Reliability was reported to be good [29]. The internal consistencies in this study were good across the three time points (0.84 ≤ α ≤ 0.86).

### Outcome measures

#### Depressive symptoms

Depressive symptoms were measured with the Dutch version of the Children's Depression Inventory [CDI; 60, Dutch version by 61]. The CDI is a 27-item self-report measure assessing symptoms associated with depression. Example items are *"I am sad"* and *"I do not like myself"*. Items were rated on a 3-point scale (1 = *never/hardly true,* 2 = *a bit true,* 3 = *very true*). The item pertaining to suicidal ideation was removed from the measure. In the analysis we used the mean score of the remaining 26 items. The reliability of the scale was reported as good [60]. In this study the internal consistencies across the three time points were adequate (0.73 ≤ α ≤ 0.75).

#### Anxiety symptoms

The Child Symptom Inventory [CSI; 62, Dutch version: 63] is a behavior rating-scale to assess childhood disorders based on DSM-IV criteria. The parent checklist was used to assess problems related to generalized anxiety. Parents rated children's generalized anxiety symptoms in the last six months on seven items (e.g., "*Is very tense or unable to relax*". Ratings were made on a 4-point scale ranging from 1 = *never* to 4 = *very often*. Internal consistency ranged from acceptable to good [62]. In this study the internal consistencies across the three time points were good (0.77 ≤ α ≤ 0.80).

#### Externalizing symptoms

The CSI was also used to assess problems related to attention deficit hyperactivity disorder (ADHD), oppositional defiant disorder (ODD) and conduct disorder (CD). Seventeen items assessed the symptoms of ADHD (e.g., *"Is quickly distracted"*), eight items assessed symptoms of ODD (e.g., *"Does things to deliberately annoy others"*) and 15 items assessed symptoms of CD (e.g., *"Has deliberately started fires"*). Parents were asked to rate each symptom on a 4-point scale, ranging from 1 = *never* to 4 = *very often*. Internal consistency has been reported to be high [29]. The internal consistencies in this study were excellent across the three time points (0.90 ≤ α ≤ 0.91) (Table 2).

### Procedure

This study was approved by the Ethics Committee of Leiden University. Written parental consent was obtained for all the participants. Parent and self-reports were completed on three measurement occasions, with 9-month intervals. The mean duration of the intervals was 9.38 months (*SD* = 0.85) between Time 1 and Time 2, and 9.93 months (*SD* = 1.13) between Time 2 and Time 3.

Participants were tested individually in a quiet room at home or at school. All questions were presented on a laptop one by one. For DHH participants, all written questions were accompanied by an optional video translation in sign language. Tests for IQ and language ability were administered at Time 2. Parents were asked to complete questionnaires online or with paper and pencil. With parents' consent, details about the participants' hearing loss were obtained from medical records.

### Statistical analyses

Statistical analyses were performed using SPSS version 25.0 (SPSS Inc., Chicago, IL, USA). Graphs were made in R version 3.6.3 (*Ggplot2* package). Correlations between study variables are reported in Appendix Table 7. Graphic representations of individual variations in the study variables over time are shown in Appendix Fig.  1. Longitudinal analyses were conducted using linear mixed models (LMMs) with maximum likelihood estimation [64]. Our data had a two-level structure with time points (level 1) nested within participants (level 2), and LMMs allow this within-participant dependency to be accounted for.

To analyze levels and developmental trajectories of emotion awareness and regulation across time, increasingly more complex LMMs were fitted to the data via a formal model-fitting procedure. We started with an unconditional means model that included only a fixed and random intercept, which was then compared to additional models that tested the grand mean trajectory of age (centered around 9 years, i.e., the age of the youngest child). We examined two age models: linear and quadratic trends. A random-slope effect for age was added to the best age model. Yet, adding the quadratic trend and random effect of age did not improve model fits, so these results are not reported here. The next model included group (0 = hearing, 1 = DHH) and the interaction between age and group, to examine whether levels and developmental trajectories differed between DHH and hearing participants.

To test whether changes in emotion awareness and regulation contributed to changes in mental health symptoms, we first calculated for each predictor variable a baseline score (Time 1) and a change score (i.e., Time 1 − Time 1, Time 2 − Time 1, Time 3 − Time 1). The baseline score was added to the model to examine the contribution to mental health of between-person differences in baseline levels of EA and ER, and the change score was added to examine the contribution of within-person changes in EA and ER. We started with fitting a LMM with control variables (age, gender, language, and group) and predictor variables (baseline and change scores), to assess the unique contribution of each predictor to the development of mental health symptoms. Next, the interactions between each predictor (baseline and change scores) and group were added to the model. Additionally, we, respectively, left out EA variables and ER variables from the model to examine if the results changed when these variables were not controlled for, which was not the case and thus are not reported here. Preferred models had significantly lower Akaike Information Criterion [AIC; 65] and Bayesian Information Criterion [BIC; 66]) values compared to simpler models.

A preliminary analysis was conducted to compare between DHH participants attending mainstream schools (*n* = 48), DHH participants attending special education (*n* = 32), and hearing participants (*n* = 227), using multivariate analysis of variance (MANOVA) with Bonferroni corrections. The dependent variables were the mean levels of the study variables, averaged across all time points. Pearson’s correlations were used to examine the association across time points between EA, ER, age, and mental health symptoms. Fisher's r to Z transformations were used to compare the strength of correlations between the groups.

Three post-hoc analyses were conducted to test whether differences between and within the DHH and hearing groups could be accounted for by socio-demographic (parental education) and developmental (IQ, language competency) variables. Using the DHH sample's median scores as the cut-off points three MANOVA tests with Bonferroni corrections were applied to examine the differences in the mean level of EA, ER, and mental health symptoms (averaged across time points) between DHH participants with high profiles (*n* = 41/44/33 for parental education/IQ/language scores respectively), DHH with low profiles (*n* = 39/36/47), hearing with high profiles (*n* = 90/147/97), and hearing with low profiles (*n* = 137/80/130).

### Missing values and multiple imputations

In this study, 63 (79%) DHH and 166 (73%) hearing participants had data at all time points. Participants with and without missing data points did not differ in age at Time 1, gender distribution, IQ, and parental education level. Yet, participants who dropped out had lower language scores than participants who attended all test sessions, *t*(252) = 2.56, *p* = 0.011. Little’s MCAR test showed that data were not missing completely at random (*p* = 0.007). Yet, the values were missing for known reasons. For example, dropouts and missing IQ and language scores were mainly due to time constraints, and hearing participants were more likely to drop out than DHH participants because DHH participants regularly visited the services where we collected the data. Thus, we assumed that the data were missing at random.

Although LMMs can account for missing follow-up data points of a participant [67], missing values in independent variables at baseline could still result in bias [68]. We thus handled the missing values at Time 1 using multiple imputations [MI; 69]), which estimates missing values based on participant characteristics and relations in the data among the participants [70]. The variables included for the estimation were age, gender, hearing status, IQ, language ability, parental education level, and parent- and self-reports. We performed ten imputations [71], and report the pooled results. An overview of missing data is shown in Appendix Table 8.

## Results

### Developmental trajectories of emotion awareness and regulation

Table 3 presents the best-fitting models explaining developmental trends of EA and ER. No developmental trends were found for differentiating emotions, bodily unawareness and communicating emotions. Regarding ER, the use of approach strategies increased with age (*b* = 0.004, *p* < 0.001), the use of avoidant strategies did not show a developmental trend, and the use of worry/rumination decreased with age (*b* = -0.002, *p* = 0.049).

**Table 3:** Regression weights (standard error) examining group differences and the developmental trajectory of emotion awareness and emotion regulation

Parameter	Emotion awareness			Emotion regulation		
	Differentiating emotions	Bodily unawareness	Emotion communication	Approach strategies	Avoidant strategies	Worry/rumination
Fixed effects						
Intercept	2.38 (0.02)***	1.89 (0.02)***	2.07 (00.02)***	2.01 (0.04)***	1.93 (0.02)***	1.93 (0.04)***
Age	–	–	–	0.004 (0.001)***	–	− 0.002 (0.001)*
Group	–	–	–	–	–	–
Age x Group	–	–	–	–	–	–
Random effects						
Variance (Intercept)	0.09 (0.01)***	0.12 (0.01)***	0.09 (0.01)***	0.09 (0.01)***	0.07 (0.01)***	0.13 (0.01)***
AIC/BIC	725.34/739.43	977.55/991.65	754.41/768.50	705.45/724.24	703.29/717.39	774.74/793.53

Group: 0 = hearing, 1 = deaf or hard of hearing. *AIC* Akaike Information Criterion, *BIC* Bayesian Information Criterion

^*^p < 0.05, ^**^ *p* < 0.01, ^***^ *p* < 0.001

### Longitudinal effects of emotion awareness and regulation on mental health

Table 4 presents the best-fitting models explaining the longitudinal relations of EA and ER with mental health symptoms. Levels of depressive symptoms were not related to age. Higher baseline levels and an increase over time in differentiating emotions (baseline score: *b* = − 0.10, *p* < 0.001; change score: *b* = − 0.06, *p* = 0.002), communicating emotions (baseline score: *b* = − 0.06, *p* = 0.006; change score: *b* = − 0.04, *p* = 0.037), approach strategies (baseline score: *b* = -0.10, *p* < 0.001; change score: *b* = − 0.06, *p* < 0.001), and avoidant strategies (baseline score: *b* = − 0.04, *p* = 0.029; change score: *b* = − 0.06, *p* < 0.001) contributed to developing fewer depressive symptoms. A lower baseline level and a decrease in worry/rumination over time (baseline score: *b* = 0.12, *p* < 0.001; change score: *b* = 0.08, *p* < 0.001) also contributed to the development of fewer depressive symptoms.

**Table 4:** Regression weights (standard error) examining the effect of emotion awareness and emotion regulation on symptoms of depression, anxiety, and externalizing behaviors

Parameter	Depressive symptoms		Anxiety symptoms		Externalizing behaviors	
Fixed effects						
Intercept	1.82 (0.11)***		1.55 (0.32)***		1.59 (0.21)***	
Age	− 0.0001 (0.0003)		− 0.0004 (0.001)		− 0.001 (0.0005)*	
Group	0.04 (0.02)**		0.05 (0.05)		0.04 (0.03)	
Gender	− 0.01 (0.01)		0.03 (0.04)		− 0.03 (0.02)	
Language	− 0.01 (0.003)**		0.002 (0.01)		− 0.01 (0.01)*	
	*Baseline*	*Change*	*Baseline*	*Change*	*Baseline*	*Change*
Emotion awareness						
Differentiating emotions	− 0.11 (0.02)***	− 0.06 (0.02)**	− 0.02 (0.06)	− 0.06 0(0.04)	− 0.01 (0.03)	0.002 (0.02)
Bodily unawareness	0.02 (0.02)	− 0.01 (0.02)	0.05 (0.05)	− 0.02 (0.04)	0.08 (0.03)*	0.04 (0.02)
Emotion communication	− 0.06 (0.02)**	− 0.04 (0.02)*	− 0.004 (0.07)	− 0.02 (0.04)	− 0.04 (0.03)	− 0.03 (0.02)
Emotion regulation						
Approach strategies	− 010 (0.02)***	− 0.06 (0.02)***	− 0.07 (0.06)	− 0.08 (0.04)*	− 0.01 (0.03)	− 0.03 (0.02)
Avoidant strategies	− 0.04 (.02)*	− 0.06 (0.02)***	− 0.06 (0.05)	0.03 (0.04)	− 0.01 (0.03)	0.03 (0.02)
Worry/rumination	0.12 (0.02)***	0.08 (0.02)***	0.08 (0.06)	0.09 (0.04)*	0.02 (0.04)	0.07 (0.02)**
AIC/BIC	− 824.88/− 735.68		485.83/572.89		− 455.09/− 368.04	

Group: 0 = hearing, 1 = deaf or hard of hearing. Gender: 0 = boys, 1 = girls. AIC = Akaike Information Criterion; BIC = Bayesian Information Criterion. Adding group interactions did not improve the models

^*^ *p* < .05, ^**^ *p* < .01, ^***^ *p* < .001

Levels of anxiety symptoms were not related to age. An increase in the use of approach strategies (change scores: *b* = − 0.08, *p* = 0.040), and a decrease in worry/rumination over time (change scores: *b* = 0.09, *p* = 0.045) contributed to developing fewer anxiety symptoms.

Levels of externalizing symptoms decreased with age (*b* = − 0.001, *p* = 0.018). Lower baseline levels of bodily unawareness (baseline score: *b* = 0.08, *p* = 0.011) and a decrease in worry/rumination over time (change score: *b* = 0.07, *p* = 0.001) contributed to developing fewer externalizing behaviors.

### Differences between and within DHH and hearing participants

No differences were found between DHH and hearing participants in baseline levels and developmental trends of EA and ER skills (Table 3). The groups also did not differ in levels of anxiety or externalizing symptoms across time, yet DHH participants had higher levels of depression compared to hearing participants (*b* = 0.04, *p* = 0.007) (Table 4). As presented in Table 4, none of the longitudinal models included interactions between group and longitudinal relations, meaning that the DHH and hearing groups did not differ in the longitudinal relations between EA, ER and mental health symptoms.

Next, the school placement of the participants was examined. Table 5 presents the results of the preliminary MANOVA test, examining group differences between hearing participants, DHH participants studying in mainstream schools, and DHH participants studying in special education, in levels of EA, ER, and mental health symptoms across time points. Findings showed a main effect for group differences (*F*(18, 592) = 3.09, *p* < *0.001*, *Wilk's Λ* = 0.79), qualified by effects for approach strategy (*F*(2, 304) = 7.64, *p* = *0.001*, *η*_*p*_^*2*^ = 0.05), and for depressive symptoms (*F*(2, 304) = 5.90, *p* = *0.003*, *η*_*p*_^*2*^ = 0.04). Post-hoc *t*-tests showed that DHH participants in special schools made less use of approach strategies and scored higher on depressive symptoms compared to the other groups. There were no differences between DHH participants in mainstream schools and the hearing participants. Descriptive statistics and results of correlation tests for the three groups are presented in Appendixes Tables 9, 10, 11. Although some significant differences in correlation sizes were detected, none of the groups was clearly differentiated from the other two groups, and, therefore, these correlations are not reported here.

**Table 5:** Results of a multivariate analysis of variance for comparing the mean levels of emotion awareness, emotion regulation, and mental health symptoms between hearing participants, DHH participants in mainstream schools (DHHm), and DHH participants in special education schools (DHHs)

Dependent variable (Mean across time points)	*F*-value	*p*-value^a^	Pairwise comparisons^b^
Differentiating emotions	0.86	0.426	
Bodily unawareness	3.89	0.022	
Emotion communication	1.54	0.216	
Approach strategies	7.64	**0.001**	DHHs < DHHm (adjusted *p* < 0.001); DHHs < Hearing (adjusted *p* = 0.003); DHHm = Hearing
Avoidant strategies	0.52	0.593	
Worry/rumination	0.23	0.795	
Depressive symptoms	5.90	**0.003**	DHHs > DHHm (adjusted *p* = 0.023); DHHs > Hearing (adjusted *p* = 0.002); DHHm = Hearing
Anxiety symptoms	1.99	0.138	
Externalizing behaviors	1.12	0.327	

*DHH-m* DHH participants attending mainstream schools (*n* = 48), *DHH-s* DHH participants attending special education (*n* = 32); Hearing: hearing participants (all studying in mainstream schools) (*n* = 227)

^a^Significance level corrected to *p* < α/9 = 0.006 for multiple analyses being conducted (in bold)

^b^ *p*-values reported here are already adjusted by Bonferroni correction

Last, the participants’ socio-demographic (parental education) and developmental (IQ, language competency) profiles were examined post-hoc. Within the DHH group, DHH children attending mainstream schools were overrepresented among the high-profile groups (72%/70%/79% of DHH groups high in parental education, IQ and language scores, respectively), while DHH children attending special schools comprised the majority of the low profile DHH groups (64%/53%/53%, respectively). Table 6 presents the results of the MANOVA tests, examining group differences in levels of EA, ER, and mental health symptoms across time points. Findings showed a main effect for language scores (*F*(27, 862.19) = 2.64, *p* < 0.001, *Wilk's Λ* = *0.79*), qualified by effects for differentiating emotions (*F*(27, 862.19) = 6.47, *p* < 0.001, *η*_*p*_^*2*^ = 0.06), for depressive symptoms (*F*(27, 862.19) = 11.01, *p* < 0.001, *η*_*p*_^*2*^ = 0.10), and for externalizing behaviours (*F*(27, 862.19) = 4.56, *p* = 0.004, *η*_*p*_^*2*^ = 0.04). Pairwise comparisons indicated that DHH participants high in language competency presented better ability to differentiate emotions when compared to both DHH or hearing participants with low language competency. Similarly, hearing participants with high language proficiency showed fewer externalizing symptoms when compared to both DHH or hearing participants with low language proficiency. Yet, when depressive symptoms were examined, a unique pattern was observed for the low linguistic-profile DHH group. While all participants with low language scores showed more depressive symptoms when compared to high-profile groups of the same hearing status (DHH-h < DHH-l; Hearing-h < Hearing-l), the low-profile DHH group scored higher on depressive symptoms when compared to all other groups, including low-profile hearing counterparts.

**Table 6:** Results of a multivariate analysis of variance for comparing the mean levels of emotion awareness, emotion regulation, and mental health symptoms between hearing and DHH participants with high or low parental education level, IQ scores, or language scores

	Parental education			IQ scores		Language scores		
*N* (DHH-h/DHH-l/Hear-h/ Hear-l)	41/39/90/137			44/36/147/80		33/47/97/130		
Multivariate test ^a^	*F(27, 862.19)* = *1.67, ****p* = *0.018****; Wilk's Λ* = *0.86*			*F(27, 862.19)* = *10.55, p* = 0*.036; Wilk's Λ* = *0.87*		***F(27, 862.19)* = *2.64, p* < *0.001; Wilk's Λ* = *0.79***		

Dependent variable (Mean across time points)	*F*	*p* ^b^		*F*	*p* ^b^	*F*	*p* ^b^	Pairwise differences ^c^
Differentiating emotions	1.45	0.230		4.53	0.004	**6.47**	** < 0.001**	DHH-h > DHH-l (adjusted p < 0.001); DHH-h > Hear-l (adjusted p = 0.028)
Bodily unawareness	2.98	0.032		2.06	0.106	1.99	0.115	
Emotion communication	1.64	0.180		1.24	0.296	2.13	0.097	
Approach strategies	3.01	0.030		1.06	0.367	1.84	0.140	
Avoidant strategies	0.95	0.416		.34	0.796	.27	0.845	
Worry/rumination	0.73	0.537		1.22	0.301	2.44	0.065	
Depressive symptoms	4.02	**0.008**	Hearing-h < DHH-l (adjusted *p* = 0.008) Hearing-l < DHH-l (adjusted p = 0.011)	4.25	0.006	**11.01**	** < .001**	DHH-h < DHH-l (adjusted *p* < 0.001); Hear-h < DHH-l (adjusted *p* < 0.001); Hear-l < DHH-l (adjusted *p* = 0.006); Hear-h < Hear-l (adjusted *p* = 0.037)
Anxiety symptoms	1.08	.357		0.79	0.498	1.26	0.289	
Externalizing behaviors	1.53	.208		1.04	0.375	**4.56**	**0.004**	Hear-h < DHH-l (adjusted *p* = 0.013); Hear-h < Hear-l (adjusted *p* = 0.010)

*DHH-h* DHH participants with a score higher than or equal to the median of the DHH sample, *DHH-l* DHH participants with a score lower than the medianm *Hear-h* hearing participants with a score higher than or equal to the median of the DHH sample, *Hearing-l* hearing participants with a score lower than the median. Pooled results after multiple imputations are reported

^a^Significance level corrected to *p* < α/3 = 0.017 for the three multivariate analyses being conducted

^b^Significance level corrected to *p* < α/9 = 0.006 for the multiple univariate tests being conducted

^c^*p*-values reported here are already adjusted by Bonferroni correction

N DHH-h / DHH-l in [mainstream / special school]: Parental education: 41[34/7] / 39[14/25]; IQ: 44[31/13] / 36[17/19]; Language: 33[26/7] / 47[22/25]

Findings also indicated a main effect for parental education which was very close to the Bonferroni correction cut-off point (*F*(27, 862.19) = 1.67, *p* = *0.018*, *Wilk's Λ* = *0.86*), qualified by an effect for depressive symptoms (*F*(27, 862.19) = 4.02 *p* = 0.008, *η*_*p*_^*2*^ = 0.04). Pairwise comparison indicated that DHH participants with a low level of parental education scored higher on depressive symptoms when compared to both high-profile and low-profile hearing participants.

## Discussion

This study employed a longitudinal design to examine the development of emotion awareness and regulation skills during adolescence and their unique contributions to the development of mental health symptoms over time in adolescents with and without hearing loss. Importantly, DHH and hearing adolescents did not differ in their baseline levels, developmental trajectories of EA and ER skills, nor in the way or strength EA and ER skills were related to mental health symptoms. Preliminary analyses suggested heterogeneity within the DHH group according to educational placement, language abilities, and parental education level, which may partly explain the differences found in previous literature. We first discuss our findings in light of previous research on DHH children and adolescents and then discuss their contribution to the field of emotion and mental health development in general.

Despite reduced access to emotion socialization and communication [4, 35], DHH adolescents did not differ in this study from hearing peers in their development of EA and ER skills. While previous cross-sectional research has found differences in ER skills between DHH and hearing preschoolers [44, 45], it is possible that some developmental gaps in ER are closed by the time the DHH kids reach adolescence, thanks to aggregated acquired experience in social interactions. Additionally, all previous adolescents' studies, which found differences between DHH and hearing peers in EA or ER [42, 46, 47], focused on participants studying at special schools for the deaf. However, children who are initially assigned to special schools often tend to have lower IQ scores compared to mainstreamed DHH peers, come from lower socioeconomic backgrounds or present additional disabilities [72, 73]. In this study as well, DHH participants enrolled in schools for the deaf presented lower levels of language scores, IQ, and parental education status, compared to their mainstreamed DHH peers (Appendix Table 9), and DHH participants in mainstream schools were overrepresented among the high-profile DHH group (in language, IQ and parental education level). Thus, school placement is likely a consequence of the students' developmental and socioeconomic profiles, and these profile differences may account to a large extent for the differences found in this study between students studying in special and mainstream schools. Preliminary analyses showed that DHH adolescents in special schools made less use of approach strategies and presented higher rates of depressive symptoms compared to the other groups. While these findings should be interpreted with caution, as they were based on exploratory analyses, it is notable that these reduced ER skills and depressive symptoms were related to profiles characterizing adolescents enrolled in special schools (see Appendix Table 11 for the correlations found in this study between approach, depressive symptoms, IQ, language skills, and parental educational status). Yet, these socio-demographic and developmental variables cannot account alone for all the differences found. Findings from post-hoc analysis indicated that while low language competency is related to higher levels of depressive symptoms, still the low-profile DHH group scored higher on depressive symptoms when compared to all other groups, including hearing participants with the same low-profile. In addition, a trend was observed for parental education, with low-profile DHH participants scoring higher on depressive symptoms when compared to both high and low-profile hearing counterparts. Possibly, personal and environmental risk factors such as low language competency, or low parental education, which is also related to low household income, exert differential impact when the child has fewer internal or external resources to rely on [74]. Such factors may further impinge upon DHH adolescents' access to communication, while at the same time the DHH adolescent has less opportunities to compensate by turning to alternative social circles. Future studies need to further explore the differential role of factors such as low socio-economic background in the formation of depression in DHH adolescents, and in the longitudinal development of emotion skills and mental health over time.

While findings suggested that developmental and socioeconomic profiles may explain the differences found between students in special and in mainstream schools, other factors may also contribute, such as an acquired sense of helplessness [43] or life-long coping with stigmatic attitudes [49]. These factors may not stem from the educational-settings themselves, but from the interaction between the personal profiles of students assigned to special education and the attitudes within their families, educational environments and the larger society. Research with DHH adolescents needs to clarify the unique contexts faced by those studying in special education beyond deafness and educational placement per-se [75] in order to better understand their emotional development and needs.

Regarding DHH adolescents who study in mainstream schools, an increasing body of research has shown that they may experience socio-emotional difficulties [e.g., reviews at 76, 77]. Our findings suggested that these children's EA and ER skills were developed to the same extent as in their hearing peers. Future research may, therefore, benefit from focusing on environmental factors at schools, such as adaptation of peers to their communication needs or social stigma [77, 78], in trying to further understand these difficulties.

The longitudinal design of this study uniquely enabled to examine the development and unique contributions of specific EA and ER strategies in light of knowledge gained from previous cross-sectional research. For all our adolescent participants, findings showed no developmental trends for EA skills and avoidant ER, an increase in approach ER, and a decrease in worry/rumination over an 18-month period. These findings partly support previous cross-sectional research [see review at 49], suggesting that during adolescence the ability to cope adaptively with emotional stress increases with age.

In line with previous studies on adolescents’ depression [e.g., 2, 16], our findings regarding EA confirmed that adolescents with an initial high ability to differentiate between emotions and to locate their antecedents, or those who showed an increase in this ability, also showed a decrease in depressive symptoms over an 18-months period. Furthermore, findings provided a first longitudinal support for the similar contribution of emotion communication with other people to a decrease in depressive symptoms [e.g., 10, 19 for cross-sectional findings]. Notably, these EA skills retained their relations to depression also after controlling for the effects of ER, thereby pointing at their unique longitudinal contribution. Unexpectedly, EA skills were unrelated to the development of anxiety, possibly because parent reports were used instead of self-reports as in previous studies [e.g., 10, 19] (see Limitations). In addition, contrary to our expectations bodily unawareness was not related to internalizing symptoms, but high baseline levels of bodily unawareness were related to higher levels of externalizing symptoms over time. While bodily unawareness was positively correlated with emotion differentiation and communication, in line with previous research (Appendix Table 7, see also, e.g., [9, 19]), our findings suggest that bodily unawareness may have a differentiated prediction with regards to the development of mental health symptoms.

Our findings provided longitudinal support to cross-sectional research on emotion regulation strategies [e.g., 13, 22], showing that high levels and increasing levels of approach and avoidance ER are related to a decrease in depressive symptoms over time. Interestingly, only an increase in approach ER was related to a decrease in anxiety symptoms. Possibly, the active stance involved in approaching the stressor prevents the generation of anxiety by increasing a sense of control over the situation. It is also possible that decisions over which regulation strategies to use are made according to the perceived controllability of the stressors [25], with perception of stressors as controllable leading both to low anxiety level and adopting an approaching stance.

As expected, worry/rumination was positively related to the development of depressive, anxiety, and externalizing symptoms over time. These findings support the strong explanatory power of worry/rumination as a core transdiagnostic factor underlying both internalizing and externalizing symptoms in adolescents [e.g., 27]. While the predictive link between worry/rumination and aggressive or disruptive behaviors has been examined so far only for boys [28, 29], this study showed that gender did not moderate this effect.

Last, emotion differentiation, emotion communication, approach and avoidance ER, and worry/rumination all contributed to the development of depressive symptoms both in their baseline levels and in their within-person change scores over time, or solely in their change scores in the case of anxiety or externalizing symptoms. These findings highlight that these emotion skills can change over time in individual adolescents and may, therefore, be subject to change. Furthermore, decreases or increases in these skills can serve as warning signs for future development of mental health symptoms and at the same time may suggest the most suitable focus for mental health interventions, benefitting adolescents with and without hearing loss alike.

## Limitations

This study examined several aspects of EA and ER which had been relatively well-researched in previous literature. However, there is a wide range of emotion skills which could yield different results and thus need further examination for their unique contributions. The sample sizes applied in this study were adequate for longitudinal comparison between DHH and hearing participants, but they did not allow for longitudinal comparisons within the DHH group. Larger sample sizes would allow for exploring in more depth the moderating role of factors such as educational placement, teachers' attitudes toward deafness, developmental factors, socio-economic background or family interactions. Qualitative methods, as well as including more DHH researchers and community members in research planning and conduction, can further enrich our understanding by 'insider' perspectives on factors influencing emotion and mental health development in different contexts. Next, levels and variance of externalizing symptoms were low for all our participants. Future studies would, therefore, benefit from assessing externalizing behaviors in larger sample sizes of various populations to confirm, for instance, the lack of a gender effect in this study on the relation between worry/rumination and externalizing symptoms. The use in this study of different informants to measure the outcomes may have reduced the power to detect associations between self-reported predictors and parent-reported outcomes, such as the lack of a significant relation in this study between EA and anxiety. On the other hand, findings question the validity of associations found when only self-reports were used. Future designs, which use multi-informants per each variable, can better examine the extent to which associations in the field of EA, ER and mental health are inflated due to common-method bias [13].

## Conclusion

This study provided a longitudinal support for the importance of several aspects of EA and ER during adolescence, by showing their unique contributions to mental health development also after controlling for each other's effects. Findings showed that decreases in certain emotion skills during adolescence might be warning signs to subsequent development of mental health symptoms. It is suggested that interventions tailored at specific emotion skills would be beneficial for prevention of distinguished mental health symptoms. Overall, findings pointed at the relative positive situation of adolescents with and without hearing loss alike, in their EA and ER development. Exploratory analyses suggested that DHH students in special schools for the deaf may be at risk for their ER and mental health development; that these differences are likely explained by specific developmental and socio-economic profiles of students assigned to special education; and that language competency and possibly also parental education level may exert a differential impact on mental health depending on the hearing status of the child. Future research needs to address the heterogeneity within the DHH population and its possible interaction with different risk factors in both cross-sectional and longitudinal designs.

## Data Availability

The dataset and associated information used in the current study will be archived on the Leiden University archiving platform DataverseNL (https://dataverse.nl/) once the manuscript is accepted. https://doi.org/10.34894/U2LD88

## Appendix

See Appendix Tables 7, 8, 9, 10, 11

**Table 7:** Pearson’s correlations between study variables across time points

Parameters	*r* coefficient for all participants (for hearing participants / for DHH participants)								
	1	2	3	4	5	6	7	8	9
1. Age									
2. DE	0.08 (0.04/.15)								
3. Bodily	− 0.05 (− 0.05/− 0.10)	0**.29** (0.**26**/0.**39**)							
4. EC	0.08 (0.08/0.06)	0.**43** (0.**41**/0.**50**)	0.**25** (0.**22**/0.**31**)						
5. Approach	0.**15** (0.**17**/0.12)	− 0.02 (0.004/− 0.08)	− 0.**14** (− 0.**11**/− 0.**20**)	0.**21** (0.**29**/0.002)^***^					
6. Avoidant	− 0.04 (− 0.10/0.08)^*^	− 0.04 (− 0.05/− 0.04)	0.08 (0.11/0.01)	− 0.04 (0.002/− 0.15)	0.01 (− 0.02/0.06)				
7. Worry	− 0.01 (0.04/− 0.10)	− 0**.44** (− 0.**42**/− 0.**49**)	− 0.**45** (− 0.**43**/− 0.**50**)	− 0.**41** (− 0.**41**/− 0.**39**)	0.06 (0.06/0.07)	− 0.**21** (− 0.**24**/− 0.12)			
8. Depression	− 0.03 (− 0.04/− 0.06)	− 0.**39** (− 0.**36**/− 0.**47**)	− 0.**13** (− 0.**11**/− 0.**23**)	− 0.**35** (− 0.**35**/− 0.**38**)	− 0.**26** (− 0.**26**/− 0.**24**)	− 0.**14** (− 0.**16**/− 0.10)	0.**42** (0.**42**/0.**47**)		
9. Anxiety	− 0.08 (− 0.10/− 0.03)	− 0.09 (− 0.06/− 0.16)	0.01 (0.01/− 0.03)	− 0.10 (− 0.08/− 0.16)	− 0.09 (− 0.**13**/0.03)^*^	− 0.04 (− 0.02/− 0.08)	0.10 (0.11/0.11)	0.**16** (0.**20**/0.06)	
10. Externalizing	− 0.**16** (− 0.**17**/− 0.15)	− 0.06 (− 0.03/− 0.13)	0.**16** (0.**15**/0.14)	− 0.**11** (− 0.11/− 0.15)	− 0.**15** (− 0.**15**/− 0.14)	0.01 (0.01/0.01)	0.02 (0.03/0.01)	0.**24** (0.**27**/0.11)	0.**64** (0.**66**/0.**60**)

*DE* Differentiating emotions, *EC* Emotion communication. Significance level was adjusted by the number of correlations, i.e., *p* < α/9 = 0.006. Significant correlations are bolded. Pooled results after multiple imputations are reported

^*^*p* < 0.05, ***p* < 0.01, *** *p* < 0.001 for the comparison of the strength of correlations between hearing and DHH participants, based on Fisher’s r-to-z transformation

**Table 8:** Overview of missing data

	DHH			Hearing		
	*N*	Missing		*N*	Missing	
		Count	%		Count	%
Time 1						
Age	80	0	0	227	0	0
Gender	80	0	0	227	0	0
IQ scores	77	3	4	199	28	12
Language scores	54	26	33	196	31	14
Maternal education level	67	13	16	164	63	28
Paternal education level	68	12	15	160	67	30
Differentiating emotions	79	1	1	227	0	0
Bodily unawareness	79	1	1	227	0	0
Emotion communication	79	1	1	227	0	0
Approach strategies	80	0	0	227	0	0
Avoidant strategies	80	0	0	227	0	0
Worry/rumination	79	1	1	227	0	0
Depressive symptoms	79	1	1	227	0	0
Anxiety symptoms	71	9	11	183	44	19
Externalizing behaviors	71	9	11	183	44	19
Time 2						
Age	78	2	3	198	29	13
Differentiating emotions	78	2	3	196	31	14
Bodily unawareness	78	2	3	196	31	14
Emotion communication	78	2	3	196	31	14
Approach strategies	78	2	3	197	30	13
Avoidant strategies	78	2	3	197	30	13
Worry/rumination	78	2	3	197	30	13
Depressive symptoms	78	2	3	198	29	13
Anxiety symptoms	59	21	26	170	57	25
Externalizing behaviors	59	21	26	170	57	25
Time 3						
Age	63	17	21	166	61	27
Differentiating emotions	64	16	20	166	61	27
Bodily unawareness	64	16	20	166	61	27
Emotion communication	64	16	20	166	61	27
Approach strategies	64	16	20	166	61	27
Avoidant strategies	64	16	20	166	61	27
Worry/rumination	63	17	21	166	61	27
Depressive symptoms	64	16	20	166	61	27
Anxiety symptoms	49	31	39	142	85	37
Externalizing behaviors	49	31	39	142	85	37

*DHH* Deaf or hard of hearing

**Table 9:** Mean scores (standard deviations) of study variables and group comparisons at each time point between DHH participants attending mainstream schools and DHH participants attending special education schools

	Mean (SD)		*t*-value^a^	*p*-value^a^
	Mainstream	Special		
Personal characteristics				
No. of participants	48	32	–	–
Age at Time 1	143.85 (20.15)	141.53 (18.58)	0.52	0.602
Language scores	10.94 (2.91)	6.5 (3.01)	3.32	**0.002**
IQ scores	10.78 (2.76)	9.28 (2.28)	2.42	**0.016**
Parental education level	3.45 (0.63)	2.8 (0.71)	3.08	**0.004**
Time 1				
Differentiating emotions	2.36 (0.47)	2.22 (0.42)	1.29	0.199
Bodily unawareness	1.89 (0.47)	2.08 (0.37)	− 1.77	0.077
Emotion communication	2.11 (0.40)	2.11 (0.33)	0.07	0.948
Approach strategies	2.24 (0.34)	1.88 (0.32)	4.86	**< 0.001**
Avoidant strategies	1.86 (0.43)	1.87 (0.36)	− 0.09	0.932
Worry/rumination	1.95 (0.50)	1.86 (.45)	0.72	0.469
Depressive symptoms	1.33 (0.20)	1.46 (0.20)	− 2.90	0.004
Anxiety symptoms	1.57 (0.45)	1.41 (0.48)	1.06	0.291
Externalizing behaviors	1.40 (0.21)	1.41 (0.20)	− 0.35	0.730
Time 2				
Differentiating emotions	2.41 (0.42)	2.34 (0.43)	0.74	0.459
Bodily unawareness	1.98 (0.47)	2.15 (0.38)	− 1.65	0.099
Emotion communication	2.08 (0.42)	2.12 (0.33)	− 0.47	0.639
Approach strategies	2.29 (.40)	1.95 (0.44)	3.49	** < 0.001**
Avoidant strategies	2.02 (0.46)	1.83 (0.39)	1.93	0.054
Worry/rumination	1.76 (0.49)	1.78 (036)	− 0.18	0.861
Externalizing behaviors	1.32 (0.21)	1.35 (0.21)	− 0.48	0.631
Anxiety symptoms	1.45 (.38)	1.43 (0.38)	0.14	0.888
Depressive symptoms	1.32 (0.19)	1.41 (0.19)	− 2.08	.037
Time 3				
Differentiating emotions	2.44 (0.48)	2.50 (0.42)	− 0.52	0.602
Bodily unawareness	1.85 (0.55)	2.13 (0.41)	− 2.17	**0.030**
Emotion communication	2.16 (0.44)	2.26 (0.34)	− 0.95	0.342
Approach strategies	2.33 (0.39)	2.09 (0.41)	2.41	**0.016**
Avoidant strategies	2.03 (0.37)	1.98 (0.33)	.48	0.629
Worry/rumination	1.76 (0.49)	1.66 (0.33)	.89	0.372
Depressive symptoms	1.29 (0.18)	1.38 (0.20)	− 1.90	0.057
Anxiety symptoms	1.52 (0.42)	1.37 (0.23)	1.24	0.217
Externalizing behaviors	1.35 (0.22)	1.33 (0.17)	0.41	0.682

Significant results are in bold

*DHH* Deaf or hard of hearing, *SD* Standard deviation

^a^Pooled results after multiple imputations

**Table 10:** Pearson’s correlations between predictor and outcome variables across time points in DHH participants attending mainstream schools (DHHm), DHH participants attending special education schools (DHHs), and hearing participants

Predictor variable	Correlation (*r*) with outcome variable (DHHm / DHHs / hearing)		
	Depressive symptoms	Anxiety symptoms	Externalizing behaviors
Differentiating	**− 0.49 / − 0.45 /− 0.36**	− 0.21 / − 0.11 / − .06	− 0.17 / − 0.02 / − 0.03
Bodily unawareness	− **0.32**^a^ / **− 0.30**^ab^ / **− 0.11**^b^	− 0.04 / 0.07/ 0.01	0.06 /** 0.34** / **0.15**
Communication	**− 0.41 / − 0.41 / − 0.35**	− 0.20 / − 0.06 / − 0.08	− **0.24**^a^ / 0.09^b^ / − **0.11**^ab^
Approach	− 0.08^a^ / − **0.26**^ab^ /− .26^b^	0.05 / − 0.10 / **− 0.13**	− 0.02^a^ / − **0.33**^b^ / **− 0.15**^ab^
Avoidant	− 0.09 / − 0.07 / *− 0.16*	− 0.16 / 0.05/ − 0.02	0.06 / − 0.07 / 0.01
Worry	**0.48 / 0.57 / 0.42**	0.11 / 0.09 / 0.11	0.06 / − 0.09 / 0.03

Significance level was adjusted by the number of correlations each predictor was in, i.e., *p* < α/3 = 0.017. Significant correlations are bolded. Pooled results after multiple imputations are reported

The character superscripts (i.e., ^a^, ^b^) denote a difference in the strength of correlations between the three groups at *p* < 0.05, based on Fisher’s *r*-to-*z* transformation. When the superscripts differ, the groups differ in the strength of correlation. When the superscripts are the same or when there is no superscript, the groups do not differ

**Table 11:** Pearson’s correlations between study variables and parental education level, language scores, and nonverbal IQ across time point

Personal variable	*r* coefficient for all participants (for hearing participants / for DHH participants)		
	Parental education	Language	Nonverbal IQ
Differentiating emotions	0.05 (− 0.03 / **0.22**)**	**0.14** (**0.12** /0.18)	**0.16 (0.14 /0.20)**
Bodily unawareness	− 0.03 (− 0.06 /0.06)	− 0.08 (− 0.04 / − 0.13)	0.01 (0.04 /− 0.05)
Emotion communication	− 0.01 (− 0.07 / .14)**	.03 (.03 / .06)	− 0.01 (− 0.01 / 0.02)
Approach strategies	0.09 (0.03 /**0.24**)**	**0.19 (0.15 / 0.27)**	0.06 (0.01 /0 .15)
Avoidant strategies	− 0.07 (− 0.08 / − 0.03)	0.02 (− 0.01 / 0.08)	− 0.02 (0.01 / − 0.09)
Worry/rumination	0.01 (0.06 / − 0.13)*	− 0.06 (− 0.06 / − 0.07)	− 0.06 (− 0.07 / − 0.06)
Depressive symptoms	− 0.07 (0.00 / **− 0.22**)**	**− 0.23 (− 0.18 / − 0.30)**	**− 0.13 (− 0.10 / − 0.17)**
Anxiety symptoms	0.01 (0.05 / − 0.08)	− 0.03 (− 0.09 / 0.11)*	− 0.01 (− 0.01 / − 0.01)
Externalizing behaviors	0.01 (0.06 / − 0.14)*	**− 0.17 (− 0.21 / − 0.05)**	− 0.01 (0.02 / − 0.05)

Significance level was adjusted by the number of correlations each predictor was in, i.e., *p* < α/3 = .017. Significant correlations are bolded. Pooled results after multiple imputations are reported

^*^*p* < .05, ***p* < .01 for the comparison of the strength of correlations between hearing and DHH participants, based on Fisher’s r-to-z transformation
